# Large air pressure changes triggered by P-SV ground motion in a cave in northern Taiwan

**DOI:** 10.1038/s41598-021-92216-w

**Published:** 2021-06-18

**Authors:** Chieh-Hung Chen, Yang-Yi Sun, Li-Ching Lin, Peng Han, Huai-Zhong Yu, XueMin Zhang, Chi-Chia Tang, Chun-Rong Chen, Horng-Yuan Yen, Cheng-Horng Lin, Jann-Yenq Liu, Ching-Ren Lin

**Affiliations:** 1 Institute of Geophysics and Geomatics, China University of Geosciences, Wuhan, 430074 Hubei China; 2 State Key Laboratory of Geological Processes and Mineral Resources, China University of Geosciences, Wuhan, China; 3 National Center for High-performance Computing, Hsinchu, Taiwan; 4 Department of Earth and Space Sciences, Southern University of Science and Technology, Shenzhen, China; 5 China Earthquake Networks Center, Beijing, China; 6 Institute of Earthquake Forecasting, China Earthquake Administration, Beijing, China; 7 Department of Space Science and Engineering, National Central University, Taoyuan, Taiwan; 8 Department of Earth Sciences, National Central University, Taoyuan, Taiwan; 9 Institute of Earth Sciences, Academia Sinica, Taipei, Taiwan; 10 Center for Space and Remote Sensing Research, National Central University, Taoyuan, Taiwan; 11 Center for Astronautical Physics and Engineering, National Central University, Taoyuan, Taiwan

**Keywords:** Environmental sciences, Solid Earth sciences

## Abstract

Acoustic-gravity waves are generally considered to be one of the major factors that drive changes of the total electron content in the ionosphere. However, causal mechanisms of couplings between sources in the lithosphere and responses in the atmosphere and the ionosphere are not fully understood, yet. A barometer in the cave of the SBCB station records an unusual phenomenon of larger amplitudes in air pressure changes inside than those at the Xinwu station (outside). Accordingly, the comparison between the recorded data at the SBCB and Xinwu station can drive investigations of potential sources of the unusual phenomenon. Analytical results of phase angle differences reveal that the air pressure outside the cave at the Xinwu station often leads air pressure changes inside at the SBCB station at relatively low frequency bands. In contrast, the larger pressure changes at frequencies >  ~ 5 × 10^–4^ Hz inside the cave at the SBCB station lead smaller changes outside at the Xinwu station. To expose causal mechanisms of the unusual phenomenon, continuous seismic waveforms are further conducted for examination. When the horizontal and vertical ground velocities of ground vibrations yield a difference in the phase angle close to 90°, coherence values between the air pressure changes and ground vibrations become large. This suggests that the pressure-shear vertical ground vibrations can drive air pressure changes. Meanwhile, the results shed light on investigating the existence of acoustic waves near the Earth’s surface using a partially confined space underground due to that the assumptions of the waves can propagate upward into the atmosphere driving changes in the ionosphere.

## Introduction

Previous studies^1–11^ reported that acoustic waves can propagate from the Earth’s surface upward to the atmosphere and drive changes in electron density in the ionosphere. However, acoustic waves originate from ground vibrations that is obscured and difficult to be identified. The difficulty is mainly caused by seismic waves generally comprising of complex vibrations. Meanwhile, excited acoustic waves disperse in an open area (i.e., near the Earth’s surface) and become weak, accordingly.

A barometer is one of the scientific instruments that is generally installed above the Earth’s surface and is utilized to monitor variations of atmospheric pressure in a particular environment. The monitoring collects useful information (i.e., atmospheric pressure) to help weather analysis and to forecast short-term changes in the weather for further evaluating impacts on human life^12,13^. Alternatively, the observation exhibits low-noise characteristics in caves and/or tunnels with rare artificial activities due to that the environment inside can mitigate influence from weather and artificial activities outside. The recorded data can be utilized as references for correcting responses of air pressure on distinct geophysical measurements^14^.

**Figure 1:**
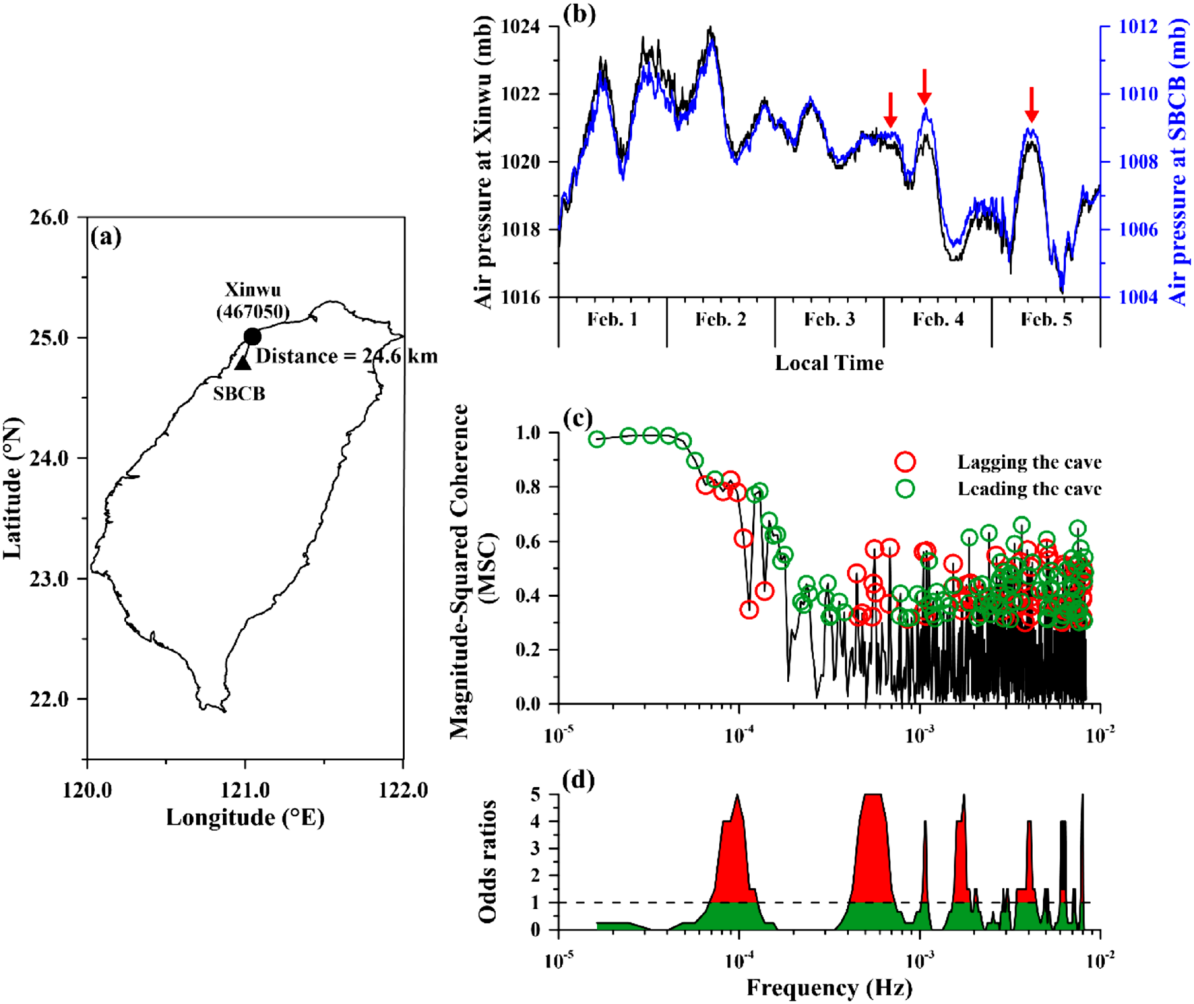
Locations and analytical results of air pressure data at Xinwu and SBCB stations. The locations of the two stations are shown in (**a**). The variations in air pressure during February 1–5 in 2016 are shown in (**b**). Black and blue lines denote the variations in air pressure at Xinwu and SBCB stations, respectively. Red arrows indicate the amplified variations in air pressure in the cave at SBCB station. The Magnitude-Square Coherence (MSC) varied with frequency that is shown in (**c**). Green (red) open circles denote changes in air pressure at Xinwu station that lead (lag) to variations in the cave at SBCB station by utilizing the phase angle at each frequency. (**d**) The statistical results of the leading values with a coherence > 0.35 determined by the odds test with a moving window of 5 events.

A cave of the SBCB station is located at (24.79°N, 120.98°E) with the altitude of ~ 141.5 m beneath the Eighteen-Peaks Mountain in the northeastern Taiwan (Fig. 1a). The cave was built for bomb shelter in 1941 (https://gps.moi.gov.tw/SSCenter/Introduce/IntroducePage.aspx?Page=Gravity4). Aisles inside the cave exhibit as a “U” sharp. The cave wall is rocks covered by calcium silicate boards and tiles. The width and the height of the cave is about 1.2–1.5 m and 1.8 m, respectively. The cave is under overburden soils with a thickness of about 40 m that causes the stable temperature and structural integrity^14^. Meanwhile, influence from groundwater is relatively small due to that the groundwater is static and its level is about 23 m lower than the cave. Doors were set up in the both entrances of the cave to avoid interference from artificial activities. A broadband seismometer was installed at the innermost of the cave (Fig. S1–S4), where is about 38 m away from the entrances, due to that the interior of the cave can eliminate the effects of the artificial activity and severe weather outside the cave. A barometer of the Setra’s Model 278 with an accuracy of ~ 0.3 mb and a sampling interval of 1 s was installed beside a broadband seismometer (Fig. S5) for correcting unwanted influence from air pressure changes on seismic data^15,16^. All the efforts are benefit to high-quality data^14^. Note that the other barometer of the Setra’s Model 270 with an accuracy of about 0.05% of the full scale and a sampling interval of 1 min was installed at the Xinwu (operated by the Central Weather Bureau in Taiwan code 467,050) weather station (25.00°N, 121.05°E,Fig. 1a) with the altitude of ~ 20 m, where is approximately 25 km away from the cave. The barometer was set a few meters above the Earth’s surface for routinely monitoring changes in air pressure dominated by the weather.

Air pressure changes inside (SBCB) and outside (Xinwu) the cave from February 1 to 5 in 2016 are plotted in Fig. 1b with the same scale (8 mb) of the Y axes for fair comparison. The air pressure outside the cave at the Xinwu station is mainly ranged from 1016 to 1024 mb. Alternatively, the air pressure inside the cave at the SBCB station is ranged from 1004 to 1012 mb. The discrepancy is considered to be contributed by difference of altitudes (121 = 141–20 m) between these two barometers based on the decrease rate (1 mb / 9 m) close to the Earth’s surface. Air pressure data show the semidiurnal variations and exhibit in-phase changes at the Xinwu and SBCB stations (Fig. 1b). Amplitudes of noise at both stations are about 0.1 mb. According to the comparison, amplitudes of the pressure perturbations at the SBCB station are roughly comparable from February 1 to 3 in 2016. In contrast, the amplitudes are approximately 0.5 mb greater than those at the Xinwu station particularly from February 4 to 5 in 2016 (red arrows in Fig. 1b as examples). The great amplitude inside the cave is an unusual phenomenon due to that artificial activities are rare, which leads us to investigate a causal mechanism of the large pressure changes at the SBCB station.

## Methodology and analytical results

We assessed the coherence of the amplitude within a particular frequency band using the Magnitude-Square Coherence (MSC) index^17^. The MSC was computed from the bivariate time series using a subroutine called “mscohere” in MATLAB. The MSC can identify significant frequency-domain correlations between two time series datasets. Meanwhile, phase angle estimates in the cross spectrum are useful for understanding where significant frequency-domain correlations exist. The differences in the phase angles of a particular frequency band were computed to understand the leading (or lagging) of air pressure changes at the Xinwu and SBCB station.

We down sampled the air pressure data retrieved from the SBCB station to a sampling interval of 1 min for fairly comparing with them obtained fdrom the Xinwu station utilizing the MSC index. The air pressure data at the Xinwu and SBCB stations exhibit coherence values close to 1 near a frequency of 3 × 10^–5^ Hz (~ semi-diurnal; Fig. 1c). The green circles at the particular frequency band in Fig. 1c reveal that variations in air pressure outside the cave at the Xinwu station occurred before (leaded) variations in air pressure inside the cave at the SBCB station. The coherence rapidly decreases and roughly maintains in a relatively-low stage of < 0.2 at frequencies > 2 × 10^–4^ Hz (without any circle mark in Fig. 1c). This suggests that the air pressure changes at these two stations are irrelevant in the relatively-high frequency band. However, the red circles lie on the coherences > 0.35 that can be frequently observed at frequencies > 2 × 10^–4^ Hz in Fig. 1c. This suggests that variations in air pressure inside the cave at the SBCB station lead them outside at the Xinwu station. The variations outside the cave lag behind them inside the cave that is entirely different from our common senses (i.e., the pressure variations should be relatively-small and quiet inside, if the variations inside are due to weather and human activity outside).

**Figure 2:**
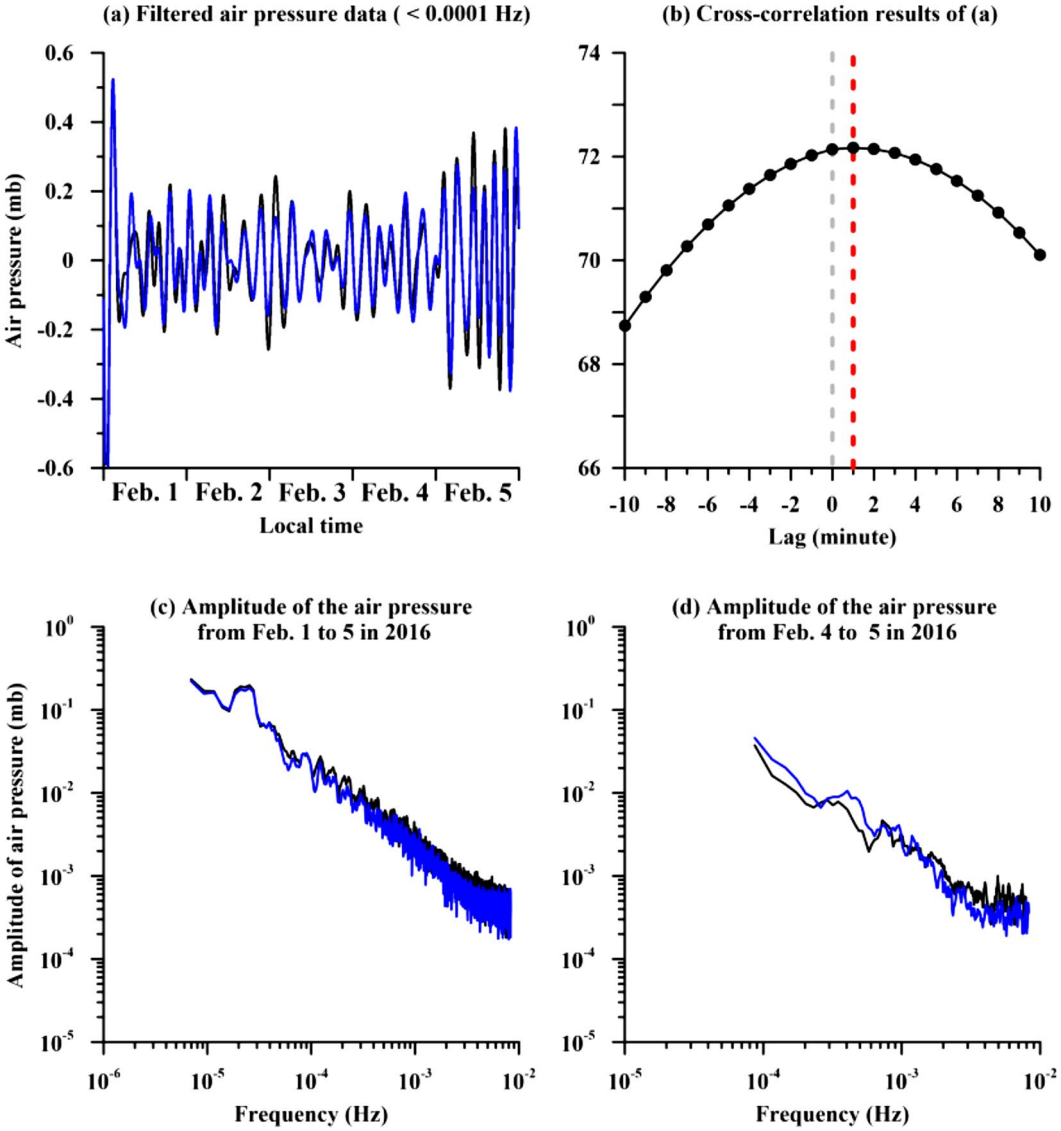
The power spectra of the air pressure data at the SBCB and Xinwu stations and their cross-correlation results in the particular frequency band. The air pressure data retrieved from two studied stations are filtered by using a low-pass filter of 10^–4^ Hz shown in (**a**). The cross-correlation results of the filtered data are shown in (**b**). The air pressure data during February 1–5 and February 4–5 in 2016 are transferred into the frequency domain shown in (**c**) and (**d**), respectively. Notably, the black and blue lines denote variations of the amplitudes with a running average of 5 continuous points at the Xinwu and SBCB stations, respectively.

We further filtered the air pressure data using a low-pass filter of 10^–4^ Hz for re-examining the coherence and the unusual phenomena. Figure 2a shows similar variations of filtered air pressure data at the two stations that is in agreement with the coherence values close to 1. The filtered data at the two stations were processed by using the cross-correlation method to estimate a time lag of them (Fig. 2b). The maximum value can be obtained when the filtered data outside the cave about one minute lead them inside. The leading is in agreement with the analytical results of phase angle in the MSC index (shown in Fig. 1c). Figure 2c shows the amplitudes of the air pressure at the SBCB and Xinwu stations in the frequency domain during February 1–5 in 2016. The amplitudes are roughly comparable at two stations due to a short distance of about 25 km. Note that the amplitude at the Xinwu station is slightly larger than it in the SBCB station, particularly in a high frequency band. This suggests that noise levels at the Xinwu station are higher than them at the SBCB station due to weather perturbations outside the cave. Figure 2d reveals the amplitudes at the two stations during the days (i.e., February 4–5 in 2016) with the unusual phenomena. Discrepancy in the amplitudes can be frequently found at frequency > approximately 4 × 10^–4^ Hz. The frequency is in agreement with it that variations of the air pressure inside the cave lead them outside determined by using the MSC index shown in Fig. 1c.

To further examine the facticity of the unusual phenomena determined from relatively-unobvious values of the coherence, the odds ratio^18–20^ is defined as *p*/(1-*p*) where *p* is the probability of success and is used in this study to show if the leading events were statistically significant. Notably, with an odds ratio near one, a success (i.e., an enhancement in this study) was more likely than a failure. We calculated the odds ratios by dividing the number of the leading events by the number of the lagging ones within a moving window of 5 events. The odds ratios are obviously larger than one in particular frequency bands (e.g., close to 1 × 10^–4^ Hz, 4 × 10^–4^–7 × 10^–4^ Hz, 1 × 10^–3^ Hz, 1.7 × 10^–3^, 4 × 10^–3^ Hz, 6 × 10^–3^ and 8 × 10^–3^ Hz in Fig. 1d). This suggests that those promising leading events can pass the statistical test (i.e., the odds ratio > 1) and exist in the observation data in the particular frequency bands. In short, we found that air pressure with an amplitude of approximately 0.5 mb at the SBCB station is larger than it at the Xinwu station. Variations of the air pressure at the SBCB station is mainly dominated by those outside the cave for the relatively-low frequency band (< 2 × 10^–4^ Hz). In contrast, for the relatively-high frequency band (> 2 × 10^–4^ Hz), variations of the air pressure at the SBCB station can lead them at the Xinwu station. The large amplitude at the SBCB station is mainly limited within the relatively-high frequency band.

## Discussions

Variations of the air pressure at the SBCB station lead those at the Xinwu station in the relatively-high frequency band of > 2 × 10^–4^ Hz (Fig. 1c). This suggests that the air can be squeezed out from the cave in the relatively-high frequency band. Even the air can be squeezed out from the cave, the pressure perturbations at the Xinwu station caused by air blowing remains questionable due to that the difference of 0.5 mb becomes smaller with the propagation via dispersion. Previous studies^5,21–23^ reported that changes in air pressure can be triggered by the arrival of propagating Rayleigh-like (Pressure-Shear vertical; P-SV) waves. Thus, beside the air blowing in the atmosphere, large-scale ground motion forces the ground and perturbs the air that can be one of the candidates for resulting the lags. The large-scale ground motion amplifies variations in air pressure changes inside the cave due to the confinement of the surrounding rocks and influences surface air pressure. In other words, relatively-large variations should result from activities inside the cave or beneath the ground that shows the possible connection between changes in ground vibrations and air pressure changes.

**Figure 3:**
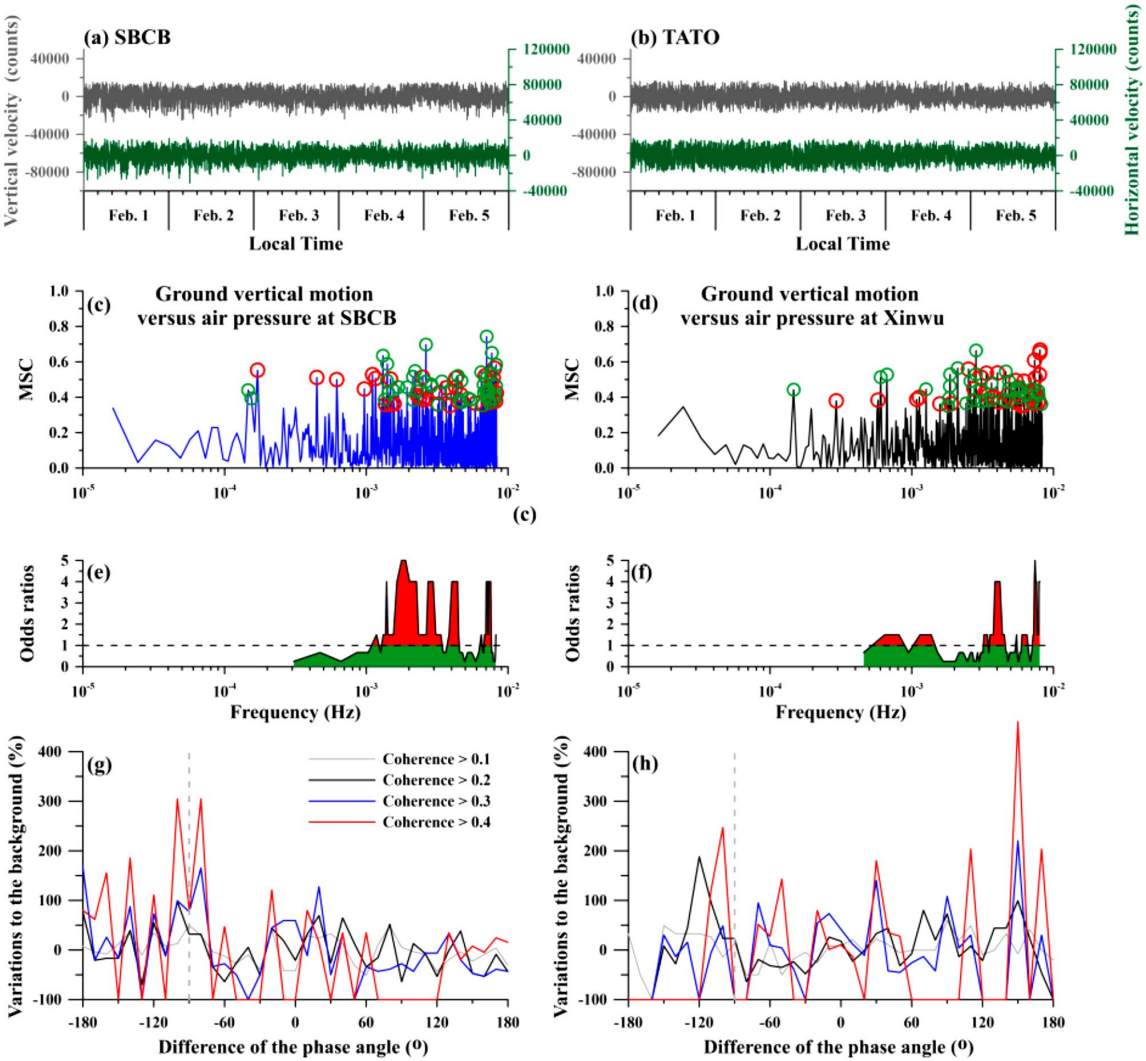
Variations in horizontal and vertical ground velocities and their comparison with air pressure data at the SBCB and around Xinwu stations. The seismic data and analytical results at the SBCB and around Xinwu stations are shown in the left and right panels, respectively. The variations in the horizontal and vertical ground velocities at the SBCB and the TATO stations, which is located close to the Xinwu station, are shown in (**a**) and (**b**). The coherence between the vertical ground velocity and air pressure at the SBCB and around Xinwu stations is shown in (**c**) and (**d**), respectively. Red (green) open circles denote that changes in ground velocity lead (lag) variations in air pressure. (**e**) and (**f**) show the odds ratios with a moving window of 5 events for a coherence > 0.35 in (**c**) and (**d**). The odds test reveals that the leading values are clustered at a few particular frequency bands. (**g**) and (**h**) demonstrate the variations of the phase angle differences for the situation with coherence values larger than the distinct thresholds at the two stations, respectively.

To examine the connection, continuous seismic waveforms (i.e., seismic data) were also analyzed in this study to understand how ground vibrations trigger air pressure variations. We down sampled the continuous seismic waveforms to a temporal interval of 1 min for fair comparison with the air pressure data (Fig. 3a). We computed the maximum horizontal amplitude as the horizontal component (Fig. 3a) by using the East–West and North–South ground velocities utilizing the method proposed by Tanimoto et al.^24^. We further computed the coherence and the phase angle difference varying with frequencies between the vertical ground velocity and the air pressure at the SBCB station in the entire study period (i.e., from Feb. 1 to 5 in 2016,Fig. 3c). A low coherence close to 0.1 in most of the frequency bands (Fig. 3c) suggests that, in a typical condition, changes in air pressure are generally uncorrelated to ground vibrations. However, the ground vibrations lead to changes in air pressure at the SBCB station (red circles in Fig. 3c), which can be observed in the frequency bands (e.g., close to 4 × 10^–4^–7 × 10^–4^ Hz, 1.5 × 10^–3^–5 × 10^–3^ Hz, 6 × 10^–3^–7 × 10^–3^ Hz in Fig. 3c,e) that exhibits the relatively-high coherences (> 0.35). Although the coherence values are not obvious, the relatively-high values suggest that changes of the air pressure in the cave are probably dominated by the ground vibrations at the SCBC station in these particular frequency bands.

We constructed a background distribution by using the phase angle differences between the horizontal and vertical components of the seismic data at the SBCB station for the situation of the ground vibrations leading changes of the air pressure with coherence values > 0. Comparison distributions are constructed by the phase angle differences for the situation with coherence values larger than the thresholds varying from 0.1 to 0.4 with a step of 0.1. The background and comparison distributions are normalized by the total number of them. The normalized background distribution is subtracted from the normalized comparison one and the obtained residual is further divided by the normalized background distribution to understand the variations of the phase angle differences to the background. No obvious characteristics can be found from the variations for the coherence > 0.1 (Fig. 3g). The variations are significant for the phase angle difference ranged between − 80° and − 100° that can be obviously observed for the coherence > 0.3 (Fig. 3g). Analytical results suggest that ground vibrations with P-SV type vibrations can trigger changes of the air pressure.

**Figure 4:**
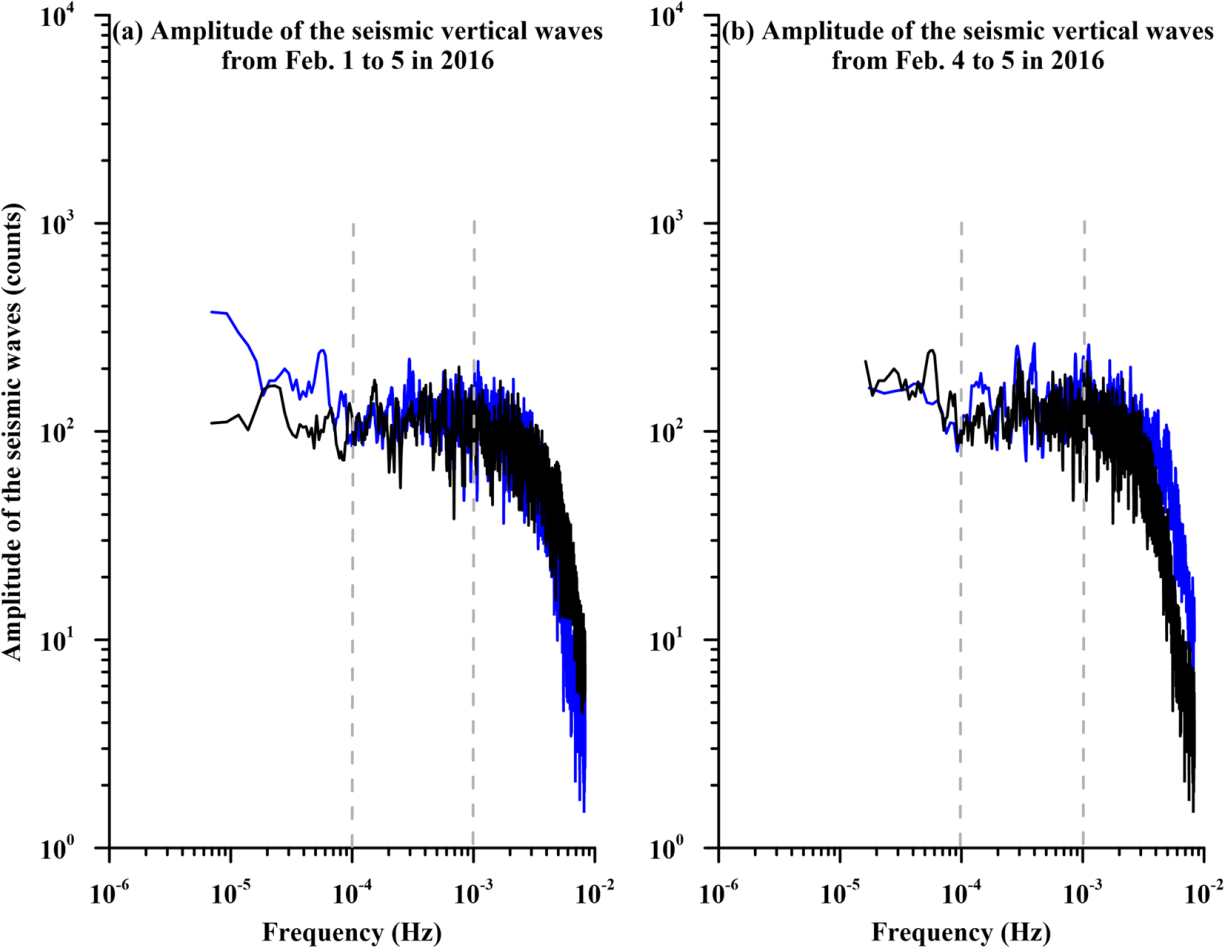
Amplitudes of seismic vertical velocities at the SBCB and TATO stations during the study periods. Seismic vertical velocities during February 1–5 and February 4–5 in 2016 are down sampled to a temporal interval of 1 min and are transferred into the frequency domain using the Fourier transform shown in (**a**) and (**b**), respectively. Notably, the black and blue lines denote variations of the amplitudes with a running average of 5 continuous points at the TATO and SBCB stations, respectively. The vertical dash lines denote the frequency at 10^–4^ Hz and 10^–3^ Hz.

We thus investigated whether air pressure at the Xinwu station changes accordingly or dissipates due to dispersion. We examined the relationship between ground vibrations at the TATO station (24.97°N, 121.50°E) retrieved from Incorporated Research Institution for Seismology (IRIS, https://www.iris.edu/hq/) (Fig. 3b) and changes in the air pressure at the Xinwu station by using the same method. Similarly, changes in air pressure at the Xinwu station are almost uncorrelated with ground motion, except for several specific frequencies close to 3 × 10^–4^–2 × 10^–3^ Hz and 3 × 10^–3^–8 × 10^–3^ Hz (Fig. 3d,f). Meanwhile, the variations to the background are significant for the phase angle differences ranged between − 120° and − 100° and at 140° (Fig. 3h). The range between − 120° and − 100° roughly yields an agreement with it observed from the SBCB station. We further computed the amplitudes of the seismic vertical velocity retrieved from the TATO and SBCB stations utilized the Fourier transform during the period of February 1–5 and February 4–5 in 2016 shown in Fig. 4. Enhancements can be roughly found in a frequency band of about 10^–4^–10^–3^ Hz during February 1–5, in 2016 (Fig. 4a). The enhancements become obvious in a wide frequency band of about 10^–4^–3 × 10^–3^ Hz during February 4–5, in 2016 (Fig. 4b). An agreement of the enhancements in the frequency band suggests that a coupling between ground vibrations in the lithosphere and variations of the air pressure in the atmosphere close the Earth’s surface.

We try to evaluate air pressure changes dominated by variations of the volume of the cave through the ideal gas law^25^. We assumed that the total number of moles and temperature of the air inside the cave are constant, while ground vibrations trigger changes in air pressure without break and/or damage. The volume of the cave in this study is approximately 270.00 (= 1.5 in width × 1.8 in height × 100 in length) m^3^. If the P-SV ground vibrations contribute variations of ± 0.25 mb in air pressure, the changes of the volume are about 0.14 m^3^, accordingly, for maintaining the product of the air pressure and the volume. If the changes of the volume are mainly contributed by the vertical component of the vibrations, the amplitude of the ground vibrations in the cave is about 10^–3^ m. The comparable results between the observation and the model suggest the large air pressure changes in a cave can be attributed to the P-SV-type ground vibrations.

**Figure 5:**
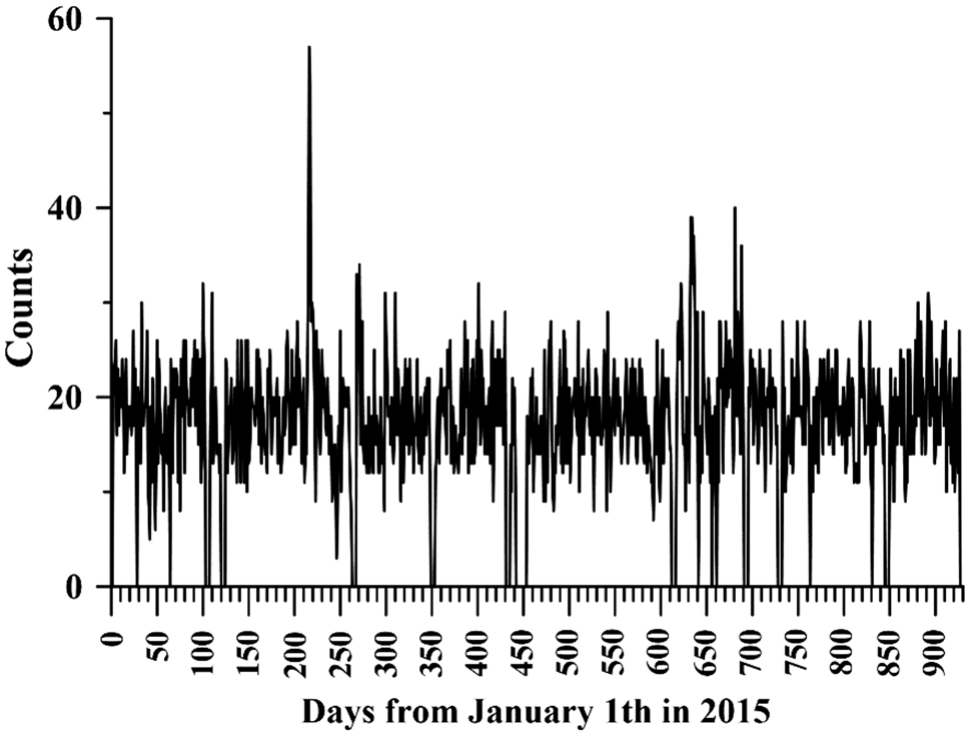
Daily counts of P-SV ground vibrations driving air pressure. The black line denotes the number of the P-SV waves at each frequency grid.

If the P-SV ground vibrations can drive changes in air pressure, the question is how often the interaction can be detected. The interaction of events by using both the P-SV ground vibrations and a coherence value > 0.3 at each particular frequency can be determined. The total number of interaction events was generally maintained at 20 during the study period of 930 days (from January 1, 2015 to July 19, 2018; in Fig. 5). This finding suggests that interactions permanently occur every day. These interactions could be dominated by P-SV ground vibrations, which are unclear and not fully understood in the world.

## Conclusion

This study proposes an efficient method to document the physical evidence of the P-SV type ground vibrations triggering changes in air pressure near the Earth’s surface. When the ground motion with the P-SV type is related to microseisms, the air pressure can change accordingly. The air pressure in caves can be amplified by the existence of the P-SV type vibrations due to the interior space being partially confined (similar to press a rubber air ball). Thus, the air pressure retrieved from a barometer inside a cave is sensitive to the P-SV type vibrations.

The novel observation sheds light on extension of the use of a cave and/or a tunnel. The amplified air pressure triggered by the P-SV type vibrations creates an excellent opportunity to study the origin of acoustic waves from ground motion. Air pressure data observed in a cave and/or a tunnel can become a treasure, while scientists want to prove the existence of acoustic waves that propagate upward and drive changes in the atmosphere. On the other hand, the P-SV type ground vibrations can be often observed in microseisms and surface waves after earthquake occurrence. When stable environments of air pressure are seriously concerned in a cave, the effects of the P-SV type ground vibrations have to be taken into consideration.

## Supplementary Information

Supplementary Information.

## Data Availability

Seismic waveform data and air pressure data at SBCB station were provided by the Institute of Earth Sciences, Academia Sinica, Taiwan. The air pressure data at Xinwu station were provided by the Central Weather Bureau, Taiwan. Those data can be downloaded at the website of https://doi.org/10.5061/dryad.05qfttdzh.
