# Supplementary material for: Large air pressure changes triggered by P-SV ground motion in a cave in northern Taiwan
Source: Sci Rep. 2021 Jun 18;11:12850. doi: 10.1038/s41598-021-92216-w (PMC8213808; doi:10.1038/s41598-021-92216-w)
Supplement: Supplementary file 1 — Supplementary Information. [file 41598_2021_92216_MOESM1_ESM.docx]

Supporting Information for

**Large air pressure changes triggered by P-SV ground motion in a cave in northern Taiwan**

Chieh-Hung Chen^1,2*^, Yang-Yi Sun^1^, Li-Ching Lin^3^, Peng Han^4^, Huai-Zhong Yu^5^, XueMin Zhang^6^, Chi-Chia Tang^1,2^, Chun-Rong Chen^7^, Horng-Yuan Yen^8^, Cheng-Horng Lin^9^, Jann-Yenq Liu^7, 10, 11^, Ching-Ren Lin^9^

^1^Institute of Geophysics and Geomatics, China University of Geosciences, Wuhan, China, ^2^State Key Laboratory of Geological Processes and Mineral Resources, China University of Geosciences, Wuhan, China, ^3^National Center for High-performance Computing, Hsinchu, Taiwan, ^4^Department of Earth and Space Sciences, Southern University of Science and Technology, Shenzhen, China, ^5^China Earthquake Networks Center, Beijing, China, ^6^Institute of Earthquake Forecasting, China Earthquake Administration, Beijing, China, ^7^Department of Space Science and Engineering, National Central University, Taoyuan, Taiwan, ^8^Department of Earth Sciences, National Central University, Taoyuan, Taiwan, ^9^Institute of Earth Sciences, Academia Sinica, Taipei, Taiwan, ^10^Center for Space and Remote Sensing Research, National Central University, Taoyuan, Taiwan, ^11^Center for Astronautical Physics and Engineering, National Central University, Taoyuan, Taiwan

**Contents of this file**

Figures S1 to S5.

**Supplementary Figure Caption**


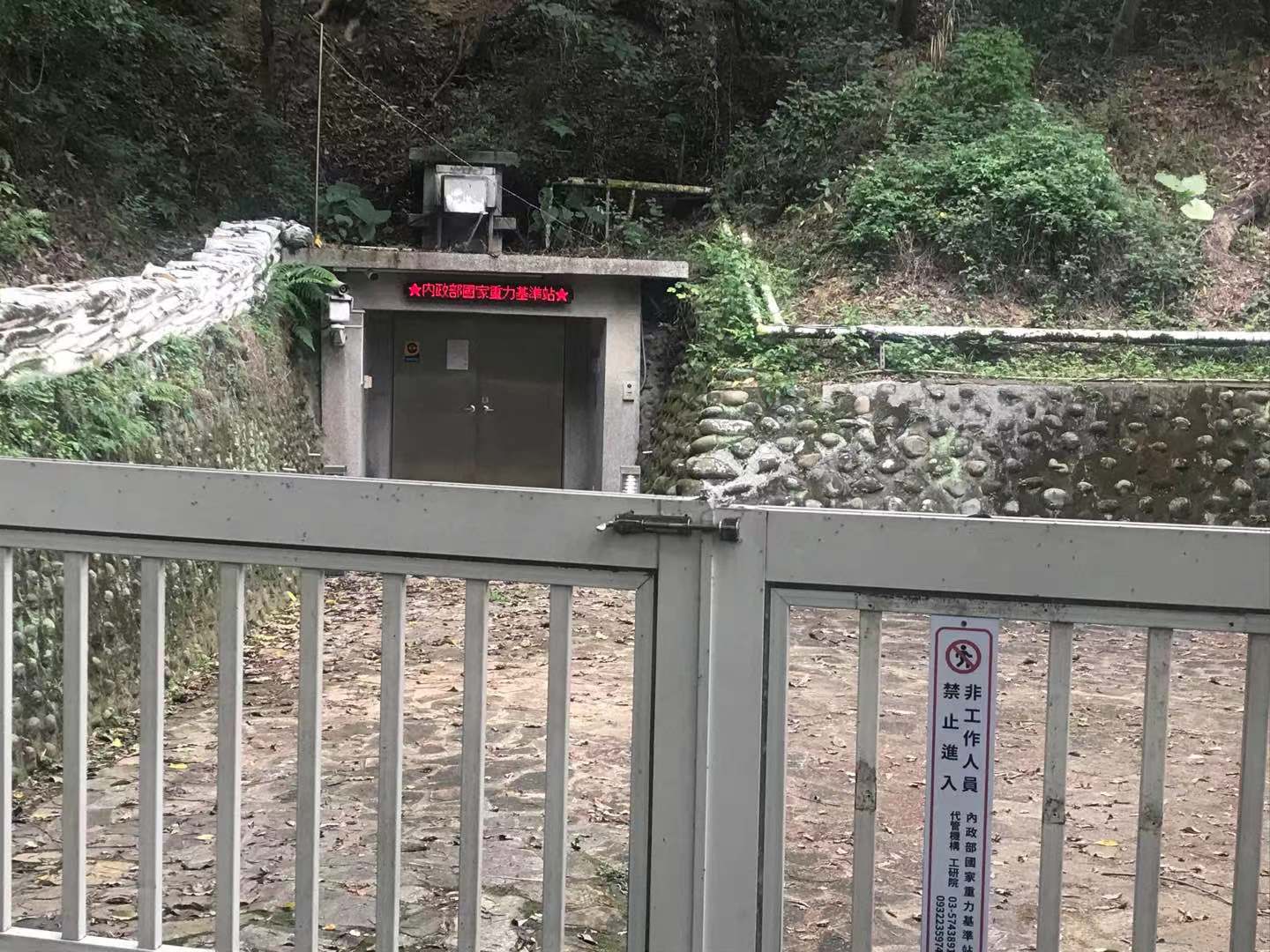


Fig. S1. The picture outside the cave at SBCB station.


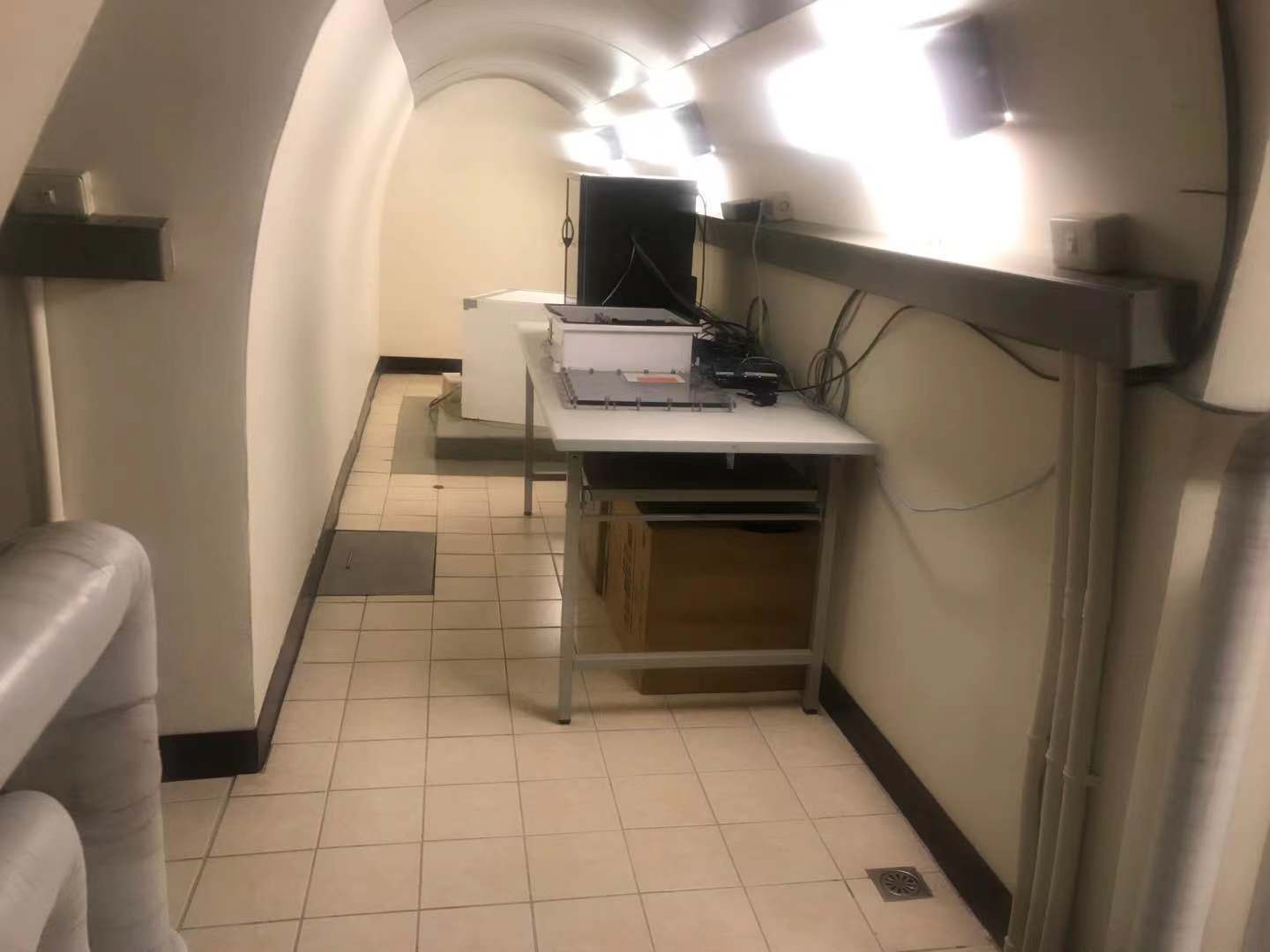


Fig. S2. The picture inside the cave at SBCB station.


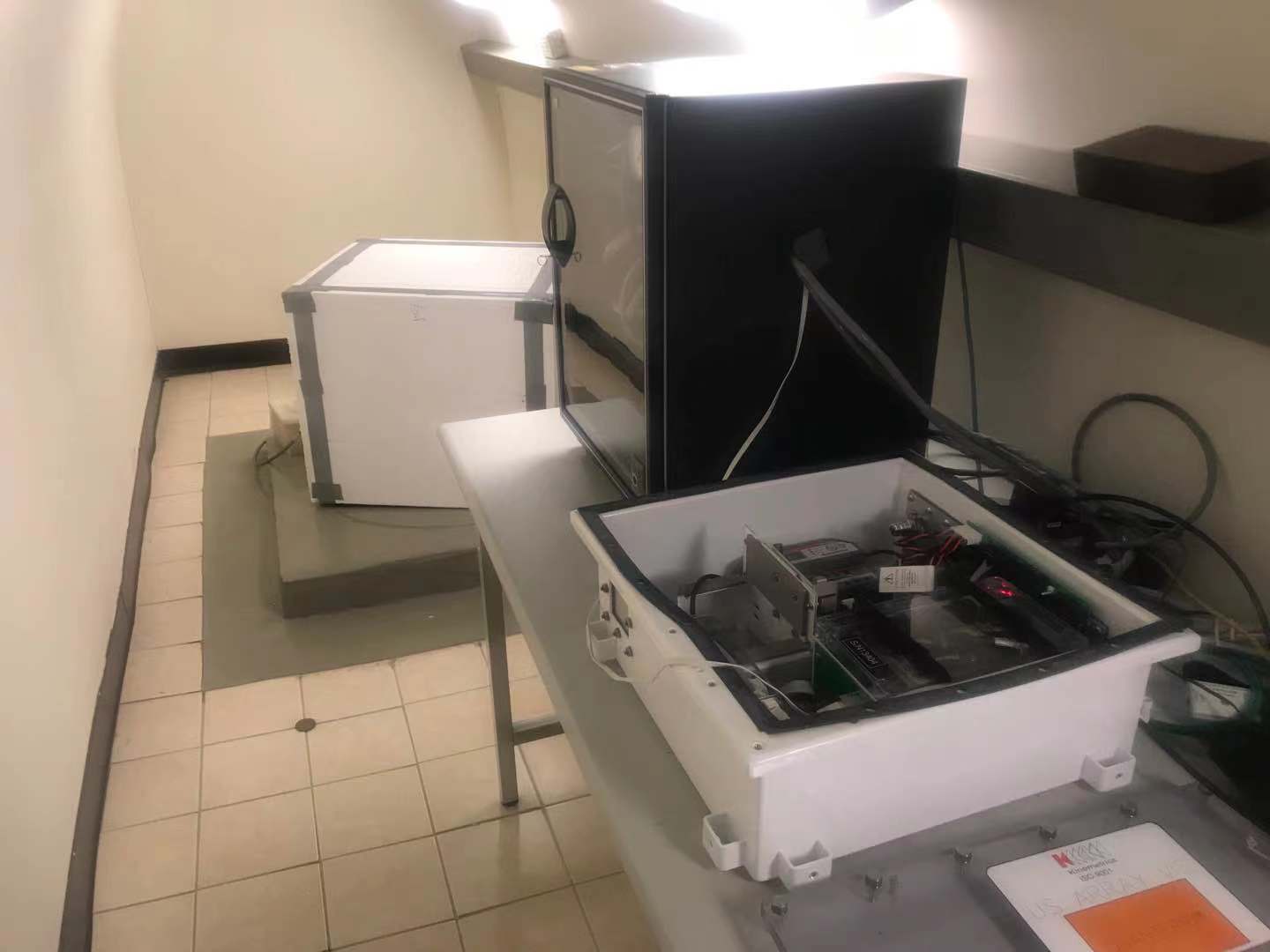


Fig. S3. The picture of the seismometer and recorders at SBCB station.


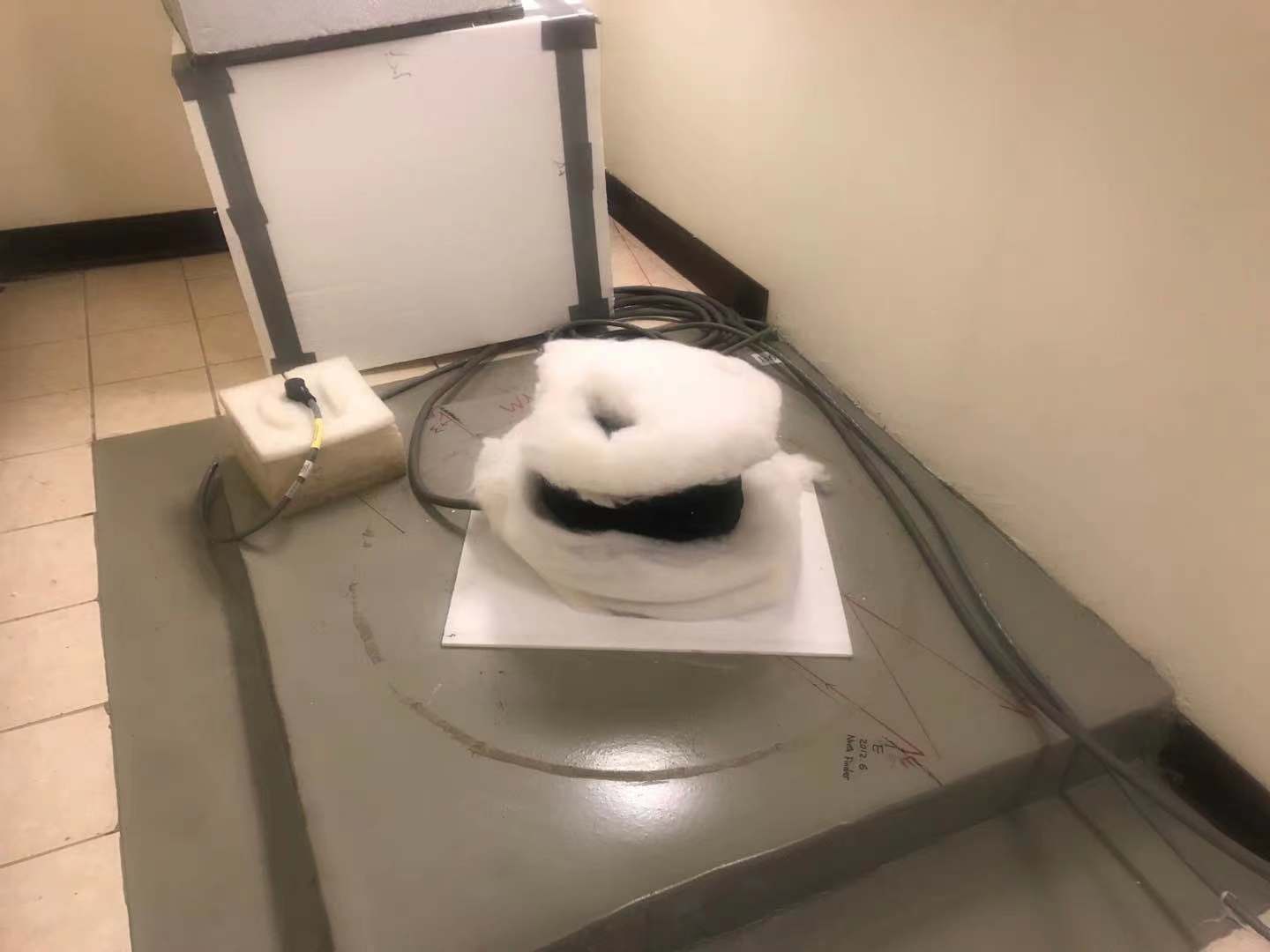


Fig. S4. The picture of the seismometer at SBCB station.


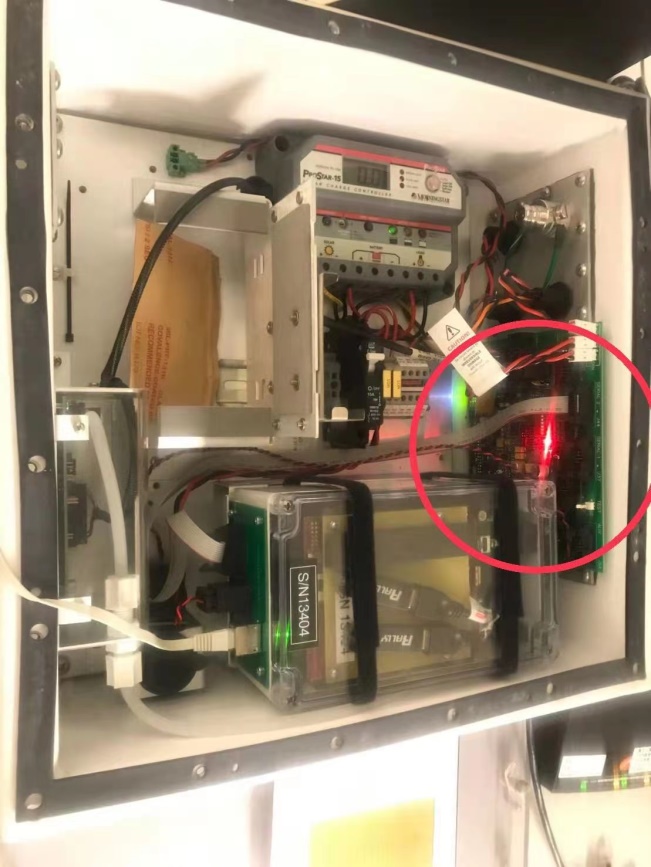


Fig. S5. The picture of the barometer at SBCB station. The barometer is marked by the open red circle.
